# Crisis as a chance. A digital training of social competencies with simulated persons at the Medical Faculty of RWTH Aachen, due to the lack of attendance teaching in the SARS-Cov-2 pandemic

**DOI:** 10.3205/zma001375

**Published:** 2020-12-03

**Authors:** Andrea Lenes, M. Klasen, A. Adelt, U. Göretz, C. Proch-Trodler, H. Schenkat, S. Sopka

**Affiliations:** 1 RWTH Aachen, Medizinische Fakultät, Aixtra Kompetenzzentrum für Training und Patientensicherheit, Aachen, Germany; 2 RWTH Aachen, Medizinische Fakultät, Modellstudiengang, Aachen, Germany; 3 Uniklinik Aachen, Klinik für Anästhesiologie, Aachen, Germany

**Keywords:** communication, Covid-19, digital training, social skills, simulated persons

## Abstract

**Objective: **The AIXTRA Competence Center for Training and Patient Safety at RWTH Aachen University has developed a concept to enable learning of communication skills with simulated persons (SP) digitally.

**Methodology: **Existing SP cases in curricular teaching were checked for digital applicability and modified. Digital seminars with the methodology of simulated conversations with SP, for planned 690 students for the courses “history taking”, 6^th^ semester, conversations in psychiatry, 8^th^ semester, and in the clinical competence course, 10^th^ semester, were conducted via video conferencing software. The structure is similar to SP-seminars in classroom teaching with a case presentation, a doctor/patient dialogue and a feedback session. In the 6^th^ and 10^th^ semester, the seminars were evaluated anonymously by the students using an online questionnaire. SP were asked by e-mail for their assessment. The lecturers were asked about their experience with the digital seminars by means of qualitative interviews.

**Results: **The survey of students with 92 completed questionnaires indicates a high level of acceptance. Digital teaching with SP was rated “very good” by 63% of the students and "good" by 37% as an overall mark for the course. The digital implementation is well practicable, the retention and accessibility of all learning goals is rated as given.

**Conclusion:** Digital teaching with SP can be well realized with appropriate preparation. Specific aspects of digital implementation (e.g. role and data protection) must be taken into account. The differentiated evaluation of the surveys will bring further results and deductive questions.

## Zusammenfassung

**Zielsetzung: **Das AIXTRA Kompetenzzentrum für Training und Patientensicherheit der RWTH Aachen hat als Konsequenz aus der entfallenen Präsenzlehre aufgrund der COVID-19-Pandemie ein Konzept erarbeitet, um das Erlernen kommunikativer Fertigkeiten mit Simulationspersonen (SP)digital zu ermöglichen.

**Methodik: **Bestehende SP-Fälle in der curricularen Lehre wurden auf die digitale Anwendbarkeit überprüft und modifiziert. Digitale Seminare mit der Methodik der Gesprächssimulation mit SP, für geplant 690 Studierende für die Kurse Anamnesetraining 6.Semester, Gespräche in der Psychiatrie 8. Semester und im klinischen Kompetenzkurs, 10. Semester, wurden über eine Videokonferenz-Software durchgeführt. Der Aufbau ist analog zu SP-Seminaren der Präsenzlehre mit einer Fallvorstellung, einem Ärztin/Patientin Gespräch und einer Feedbackrunde gestaltet. Die Seminare wurden im 6. und 10. Semester per Online-Fragebogen anonym durch die Studierenden evaluiert. SP wurden per Mail um ihre Einschätzung gebeten. Die Dozierenden werden mittels qualitativer Interviews zu ihrer Erfahrung mit den digitalen Seminaren befragt.

**Ergebnisse: **Die Befragung der Studierenden mit 92 ausgefüllten Fragebögen weist auf eine hohe Akzeptanz hin. Die digitale Lehre mit SP wurde von 63% der Studierenden mit „sehr gut“ und von 37 % mit „gut“ als Gesamtnote für den Kurs bewertet. Die digitale Durchführung ist gut praktikabel, die Beibehaltung und Erreichbarkeit aller Lernziele wird als gegeben eingestuft.

**Schlussfolgerung: **Digitale Lehre mit SP ist mit entsprechender Vorbereitung gut realisierbar. Spezifische Aspekte der digitalen Durchführung (z. B. Rollen- und Datenschutz) müssen beachtet werden. Die differenzierte Auswertung der Befragungen wird weitere Ergebnisse und ableitende Fragen bringen.

## Background

Due to the COVID-19 situation there was no classroom teaching at the medical faculty of RWTH Aachen University in the summer-semester 2020. The AIXTRA Competence Center for Training and Patient Safety has developed a concept to maintain the learning and training of social competence and communication in a digital format. 

There is little research on “digital conversations with simulated persons (SP)”. This is remarkable considering the international experience with telemedicine [1]. Also, the concept of “telesimulation”of digitally supported teaching and learning is mostly limited to the acquisition of psychomotoric skills using simulation models [2]. Approaches to digital SP teaching do exist, but have not been well researched so far. So-called virtual SP should be mentioned here. Apparently, some skills can be successfully trained with virtual SP [3], but the participants often perceive the settings as artificial and unsuitable for communication training [4]. There is evidence that communication training via video call works as well as presence-SP-training. In particular, satisfaction with the conversation, perceived information exchange and the development of an interpersonal relationship appear to be similarly good [5]. A new review [6] shows that both the medical quality and patient satisfaction seem to be similar to direct conversations. 

## Method

The implementation took place in the curricular program for 690 students for the 6^th^ semester anamnesis course (12 seminars), the psychiatry communication course, 8^th^ semester (20 seminars) and the clinical competence course 10^th^ semester (14 seminars). Participation was, like all curricular courses in the summer-semester 2020, voluntary. The development took place in a multi-level program.

1. Review and modification of roles and learning objectives for digital feasibility using video telephony via zoom
2. Telephone enquiries about the SP's readiness and technical equipment
3. Preparation of the SP: topic role protection, privacy, discarding the role
4. Technical trial run
5. Rehearsal with simulated participants in the form of student assistants of the Aixtra
6. Continuous improvement through iterative process

The digital setting is analogous to the presence teaching with SP: The lecturers and the observing students can hear and see the conversation partners, but are not visible themselves. The subsequent peer feedback takes place with the visual participation of everyone. The SP remains in Zoom's “waiting room” during the briefing. For the SP conversation the host mutes all students until the feedback round. Students are informed before the start that the conversation is subject to confidentiality and that recordings are not allowed. The RWTH has developed a privacy policy for Zoom use, which is provided to students in advance. 

Students were able to give a rating voluntarily and anonymously after participation. 19 statements about content, organization and technology of the seminar could be rated on a 6-step Likert scale. The SP were asked about their assessment by e-mail. A survey of the lecturers in the form of qualitative interviews is currently being conducted.

The study protocol was submitted to the Ethics Committee and approved by them with EC number 185/20.

## Results

In the meantime, a total of 46 seminars with 2-3 cases and approx. 15 students per seminar were conducted in the 6^th^, 8^th^ and 10^th^ semester, 26 of which were evaluated by means of questionnaires. Oral feedback was asked for in the 20 psychiatry sessions.

The evaluation of the questionnaires showed a response of n=92, with 390 possible participants. 

In the school grading system, 63% gave a “very good” and 37% a “good” as an overall grade for the course. “I can recommend the course to others” was rated “fully applicable” by 64.13% and “applicable” by 22.8%. “The cases worked on were realistic” was evaluated by 73.36% as fully applicable and 20.88% as applicable.

Potentially critical points of the digital SP seminars can also be identified. Occasionally it seems to occur that students dial in, but are not present at the computer or smartphone during the seminar, which is not visible to the lecturers because the camera is switched off. Such a “dummy presence” can particularly disturb the feedback round when students are asked to give feedback and no reaction is received.

Regarding the SP, a positive attitude towards digital teaching dominates descriptively.

As a conclusion, it can be stated that the selected cases are well feasible from a technical point of view as well as in terms of learning goals for those students who participate reliably. The situation should be trained with the SPs before starting. Thereby, emphasis should be placed on both communicative and technical aspects.

## Summary and outlook

Within a short time a digital teaching concept for the curricular teaching with simulated persons could be developed and implemented. The result was a well-founded and practicable concept with accompanying evaluation. 

The seminars were successfully conducted in terms of technology, organization and content. In the video conferences, the students show a high level of acceptance and willingness to participate and express themselves predominantly positive. 

The differentiated evaluation of the questionnaires will provide more precise information about the satisfaction of the students, the SP and the lecturers and lead to the deduction of further questions. In case the format of the “telemedical consultation” will be established, it is planned to adapt the SP's roles to the setting in terms of content.

## Competing interests

The authors declare that they have no competing interests. 
